# QAnon, authoritarianism, and conspiracy within American alternative spiritual spaces

**DOI:** 10.3389/fsoc.2023.1136333

**Published:** 2023-06-21

**Authors:** Christopher T. Conner

**Affiliations:** Department of Sociology, University of Missouri, Columbia, MO, United States

**Keywords:** QAnon, conspirituality, culture industry, sociology of religion, cultural appropriation, authoritarianism, Frankfurt School

## Abstract

**Introduction:**

QAnon, in the United States, has become something of household name due to its role in the January 6th insurrection, and because of the relatively high degree of media attention it has received. While such coverage has been useful in understanding this conspiracy movement, it has also painted a picture of QAnon that is incomplete.

**Methods:**

Using a qualitative ethnographic approach I analyzed 1,000 hours of QAnon content produced by 100 QAnon influencers. I created a database of 4,104 images (tweets, screenshots, and other static forms of communication) and 122 videos.

**Results:**

We found three separate cultural entry points not typically associated with the movement—Yoga and Wellness Groups, Neo-Shamanistic circles, and Psychics. By colonizing these spaces QAnon was able to embed itself, disguise its abrasive features, and go largely unnoticed by the general public.

**Discussion:**

This study reminds us that authoritarianism can take root in a variety of spaces, and that within each of us lie potentially fascistic tendencies—even those seeking enlightenment, through alternative practices.

## Introduction

QAnon has been described by the media and academics as a far-right extremist “big tent” conspiracy theory. Its name stems from its leader Q, who is believed to be a high-ranking government official with Q-Level security clearance, who posts information on a secret ongoing war between a cabal of pedophilic ruling elites. Believers in the movement also hold that Donald J. Trump has been ordained by God to liberate the world from these ruling elites. The movement holds that individuals such as celebrities, wealthy liberal elites (i.e., Bill Gates and George Soros), Democrats, and prominent Jewish figures harvest the blood of children as part of a Satanic ritual. Adherents to the movement claim that members of the cabal harvest the adrenal glands of children to produce a regenerative drug known as adrenochrome. Researchers of far-right groups have traced the lineage of these ideas back to a centuries old piece of Russian antisemitic propaganda first published in 1913, and later used by the Nazis to indoctrinate German youth to these ideas (Echo, 2005; Eisner, 2005). The movement achieved notoriety due to members' presence at the January 6 Capitol riot, politicians in the United States repeating or somehow endorsing the conspiracy theory, and the often violent actions of members, including multiple murders (Hernandez, 2021).

**Figure 1:**
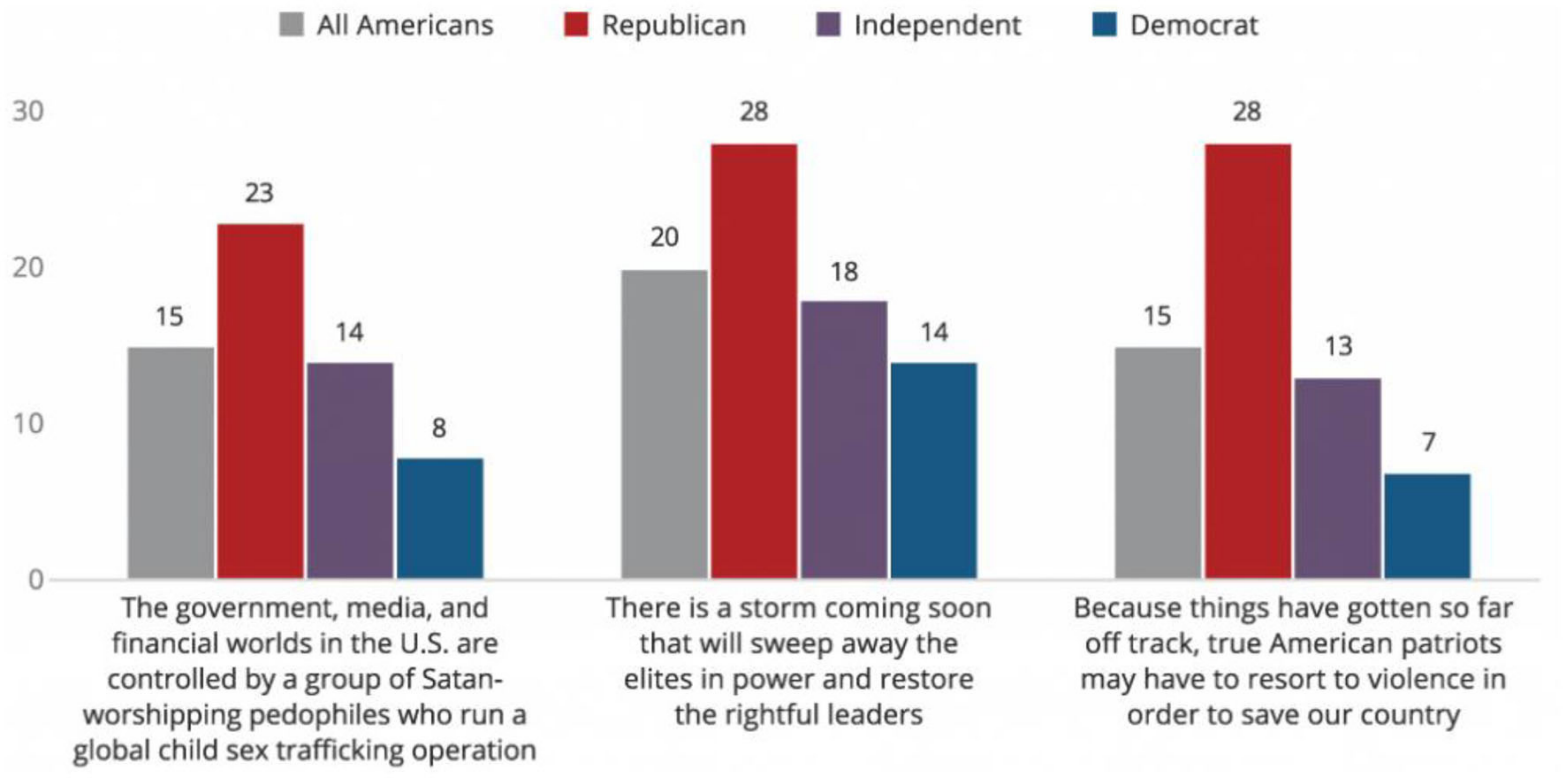
QAnon conspiracy theories by party affiliation (https://www.prri.org/research/qanon-conspiracy-american-politics-report/).

While most attempts to understand the demographics of the QAnon movement concur that it is predominately made up of Republicans, there is also a sizeable number of those who identify as Democrats who also believe in the movement (see Figure 1). A recent survey of 5,625 Americans by the Public Religion Research Institute found that 8% (*N* = 141) of Democrats and 14% (*N* = 244) of independents surveyed believed the statement that “the government, media, and financial worlds in the U.S. are controlled by a group of Satan worshiping pedophiles who run a global child sex trafficking operation” (PRRI, 2021). Together, independents and democrats make up nearly the same amount as Republicans who also believed in the statement, at 23% (*N* = 381). This important data point suggests that, despite media framing and the potential for stereotypes to emerge, belief in QAnon is not restricted to one particular group but can find an audience in a variety of different cultural settings. However, as I will show, the particular form under which QAnon appeals to these different groups is considerably different (Dickson, 2020; Kelly, 2020; Roose, 2020). In fact, as some QAnon researchers have reported, were it not for many of these “alternative lifestyle groups” typically associated with more leftist political practices, it might not have had such a significant impact (Argentino, 2021; Crockford, 2021a,b; Pace and Devenot, 2021).

### Extremism, online culture, and conspiracism

The white power movement in the United States has been rapidly evolving over the last decade, remaking itself into an aesthetically pleasing, more sophisticated appearing movement (Simi and Futrell, 2015; Southern Poverty Law Center, 2017; Miller-Idriss, 2020). One of the primary forces allowing for this rebranding is the role of the internet, social media, and anonymous online forums such as the message board 4chan (Beran, 2019; Amarasingam and Argentino, 2020; Argentino, 2020). Within these dark recesses of the internet the far right found a foothold in what might have been otherwise benign communities of young, predominately male youth (Gray et al., 2016; Gray, 2020). By using dog whistles, irony, and playing into the toxic masculine culture that existed within these communities, the alt-right was able to grow in new ways. Despite increasing calls for research exploring the impacts of social media and the internet, our knowledge of online social movements is mostly informed by our understanding of progressive movements (Tufekci, 2017). Only a handful of researchers have researched social media's role in the development of the far right (Gallagher et al., 2021; Hannah, 2021).

Additionally, as has been well documented by researchers studying extremism online, social media and other tech companies utilize powerful algorithms that incentivize content creators to produce media that is increasingly more extreme in the claims made (Gottschalk, 2019; Barbera, 2020; Hannah, 2021). Initially, alt-right groups targeted online gaming platforms and embedded Neo-Nazi imagery into those spaces (Gais and Hayden, 2020). According to some independent reporters (Kim, 2022; Philion, 2022), the goal was to push right-wing propaganda into the mainstream while also enriching themselves. There are also other examples in which online communities, such as the message board 4chan, hijacked existing culture and reappropriated it for nefarious purposes (Colley and Moore, 2020).

## Presence of QAnon in alternative spaces

QAnon consists of an elaborate and broad network of people, ideas, and interactions that we argue has transitioned into something similar to a social movement or new religious movement (Miller-Idriss, 2020). This broadness stems from its self-described label as a “big tent conspiracy movement”. This means the movement includes a wide range of beliefs, such as those who believe the earth is flat, listeners of far-right conspiracy theorist Alex Jones who reframes nearly every historical event as a government plot (i.e., school shootings, 9/11, and COVID-19), and even those going on spiritual retreats using Ayahuasca to find resonance with QAnon (Holman, 2011; Pace and Devenot, 2021; Weill, 2022). Indeed, attending QAnon rallies one will find artifacts (i.e., bumper stickers and t-shirts) and conversations on these topics which then bleed over into support for the Make America Great Movement (MAGA) started by Donald Trump. This notion of ideological mixing of ideas has been discussed at greater length by Barkun (2013) who calls this stigmatized knowledge—disproven collections of beliefs in which one is stigmatized for clinging to them. Barkun (2013) predicted the intertwining of religious belief, political ideology, and conspiracism that has given rise to the fantastical, if not fascist, millenarianist movement known as QAnon.

## The culture industry

Fascist movements dating back to the Nazis have a long history of co-opting popular cultural movements in order to gain membership, draw widespread attention, and to normalize talking about ideas couched in racist ideology (i.e., questions and debates over who really controls society are little more than anti-Semitic dog whistling). This concern was part of what drove the Frankfurt School's research, especially on the culture industry which they saw as highly important for Hitler's rise to power (Horkheimer and Adorno, 1972 [1944]; Held, 1980; Kellner, 1989). One of the early strategies in mid-eleventh century Germany was the creation of the Aaratman League, a back to nature movement that advocated for a sense of German pride through environmental protection—tapping into both a sense of national pride and a romanticization of rural life (Williams, 2007). Other similar groups also emerged to rewrite historical narratives, and appropriated occult and other esoteric practices in order to justify their beliefs (Goodric-Clarke, 2003).

Today we see similar signs of this appropriation including the creation of white power music scenes, fitness and wellness groups, and within other cultural practices not typically associated with far-right ideology (Simi and Futrell, 2015; LeClerc, 2022). This paper looks at how far-right groups appropriated the wellness industry (i.e., the creation of yoga and spiritual tourism) (Holman, 2011; Bowers and Cheer, 2017). These fields sell “eastern” mysticism to clients far and wide seeking to “re-enchant” their everyday lives, resolve problems that are the result of living in an alienated society under austerity and extreme wealth inequality, and give their lives meaning (Berman, 1981; Ward and Voas, 2011; Parmigiani, 2021; Peters, 2022). Researchers describe this as conspirituality, a word splice of conspiracism and spirituality (Asprem and Dyrendal, 2015; Chia et al., 2021; Beres et al., 2023). Outside of the context where these practices emerged, injected with Western ideology that saw them as tools to achieve success (financial or otherwise), and sold to consumers as products to fix their lives, these groups became re-oriented to facilitate the rise of right-wing ideas.

Because of its reliance on media, QAnon relates to another concept introduced by the Frankfurt School—The Culture Industry. As a theoretical concept, the culture industry, was first developed in the 1940s in the work of Frankfurt School critical theorists, Horkheimer and Adorno (1972 [1944]). In the introduction to a later article, Adorno (1975) points out that the term was developed as a critical response to the notion of “popular culture”, which implies that culture emerges spontaneously from the people. Instead, the culture industry, they argued, is imposed from the top down, or administered. However, Horkheimer and Adorno's contemporaries understood that authentically produced culture could be co-opted (Schlembach, 2015).

The term culture industry consists of two main features: standardization and pseudo- individualization. Standardization, Horkheimer and Adorno argued, was made possible by new technologies of mass communication (in their time, the TV, radio, and film). These developments in turn allowed for the application of Fordist-style production techniques that made possible the quick, cheap, and profitable reproduction of cultural commodities. Thus, a sphere of life where economic factors were once relatively minimal is now shaped by them as a primary motivation. The result, according to Horkheimer and Adorno, is a cultural sphere devoid of critical thought, and ideologically harmful to consumers by dulling their intellectual capacity. Moreover, they were concerned that this would open the doors to authoritarianism and to fascism.

The second feature of the culture industry, pseudo-individualization, refers to the notion that individual cultural products, be they works of art, music, literature, or film, are marketed as unique when, in fact, they are all derived from a common formula. In other words, the culture industry employs derivatives to make individual meaningful connections by exploiting collective emotional states. It also appeals to different market segments through the creation of different genres. Lowenthal (1949) and Adorno (2000) observed in their analysis of the radio its potential to unlock fascist tendencies; I see live streaming and other forms of internet content creation as utilizing similar social psychological techniques to manipulate consumers. However, unlike the radio, this new form of media is slightly more powerful due to the interactive nature that can be created between producers and consumers. In this way, QAnon content creators and influencers were able to exploit the inherent ways in which this new medium allows for such connections to be employed^1^.

## The authoritarian personality

Authoritarianism is typically thought of as a unified and well-defined concept (Costello et al., 2021; Satel, 2021), however Adorno and his contemporaries recognized that even self-identified “leftists” could become oriented toward authoritarianism (Adorno et al., 1950). This observation has created a variety of debates centered around whether or not anyone could be considered “leftist” and authoritarian (see Stone, 1980), however the field of extremism understands this as a process that occurs gradually over time through exposure to increasingly authoritarian ideas (Lipset, 1959; Stern, 2019). Furthermore, Adorno's study revealed that even artists could espouse similar views that aligned them with the those expressing authoritarian ideas (Adorno et al., 1950, p. 781). This may be the reason why members of the Frankfurt School were so critical of the anti-war movement (Schlembach, 2015). If we are to take the Frankfurt School's work seriously, monitoring the non-normative ways in which authoritarian spreads is important for advancing our understanding of the phenomenon (de Regt et al., 2011).

Other contemporary studies in authoritarianism have made several advances in the study of the concept since Adorno's era. Altemeyer (1996), for example, has offered a much more manageable measurement system that looks at only three of the seven traits outlined by Adorno: aggressiveness, submissiveness, and conventionalism. Altmeyer's critics note that even this modified scale produces an unmanageable amount of data (Smith, 2019). These same critics note that authoritarianism is best understood as a wish for order and stability, which tend to be better predictors (Smith, 2019). Even still, the image that alternative religious beliefs typically conjure seems incompatible with authoritarian ideology—something this paper attempts to dispel, and one of the major points made by Adorno et al. (1950).

One of the defining features of youth subcultures is the rejection of what they perceive to be “mainstream” ideas and a subversion of authority (Heanfler, 2013; Conner and MacMurray, 2022). Work by Smith and Gunn (1999), and later Smith (2018), note the power of aggression, and aggressive tendencies, within authoritarianism. Thus, the presence of authoritarianism and fascist tendencies within sub-groups not typically associated with those ideologies. This may be the result of alt-right groups tapping into the countercultural tendencies within such groups. By doing so, authoritarian leaders thus tap into the emotional energy and resentment felt by members who feel as though societal institutions no longer represent their interests. It also explains why those who in a previous generation would be considered “hippies” or part of “the counterculture” could be persuaded to see Donald Trump as a leader.

## Methods

I first began in 2019 by monitoring different Facebook groups, Instagram channels, and investigative journalists writing about QAnon. Due to lockdowns associated with the pandemic, I relied heavily upon a thematic analysis research design and participant observation of QAnon communities to inform this study (Conner, 2019, 2023; Conner and Dickens, 2023).^2^ One might be inclined to call this study design a digital qualitative ethnographic approach, in which my focus is on those who produce online content, and creating online communities related to QAnon. To date I have watched ~1,000 h of video and audio produced by QAnon influencers, those critical of QAnon (i.e., the QAnon anonymous podcast and other investigative journalists), monitored QAnon-related hashtags, collected mainstream journalistic reports on the movement, observed interactions on the GreatAwakening.win message board, monitored telegram channels used by QAnon influencers, and attended (virtually) QAnon-related events that I could live stream.^3^

My sample of individuals consists of those making content related to QAnon. Specifically, my team of research assistants tracked and monitored ~100 QAnon influencers. We collected 4,104 images (tweets, screenshots, and other static forms of communication) and 122 videos. As an online subculture there is no directory of participants, no catalog, registry, or phone book from which we could pull a systematic analysis—they are a hidden population (Hekathorn, 1997; Adler and Adler, 2003). The avenue that I chose to resolve this was to analyze the content created by QAnon influencers. By looking at the content produced by and about QAnon movement, I was able to gain insight into the ideological system that makes up the “movement”. There is also a long tradition in sociology of this kind of observational research, especially in the field of deviance, where research on certain “hidden” populations becomes problematic (Polsky, 1967; Hekathorn, 1997).

As this study continued, it became more difficult to find data on the subject because of companies like Twitter, Facebook, Reddit, and others who began to censor information (Sangeet, 2019) related to QAnon—this started before the storming of the United States' capitol on January 6, 2020 and has increased since then. Instead, I relied upon influencers making content on the various websites and spaces devoted toward QAnon. Like Goffman's (1979) study of gendered ads, I used the material on the movement itself.^4^ My first initial analysis was to try and establish the characteristics and beliefs of the movement. The second wave of analysis attempted to categorize the different strands of QAnon, with an eye for variations of the conspiracy theory.

During the course of my observations, I quickly observed that there were multiple branches of QAnon. There was a “mainstream” branch of QAnon, but there were also other branches of the movement which did not fit dominant narratives about the subculture. An additional quagmire was that many of the individuals within yoga and wellness groups, alternative spiritual groups, and psychics that promoted QAnon often refer to themselves as having been, or currently being, “left”.^5^ One of the first observations to draw my attention to this were messages supporting QAnon on message board devoted to the jam band Phish.^6^

**Figure 2:**
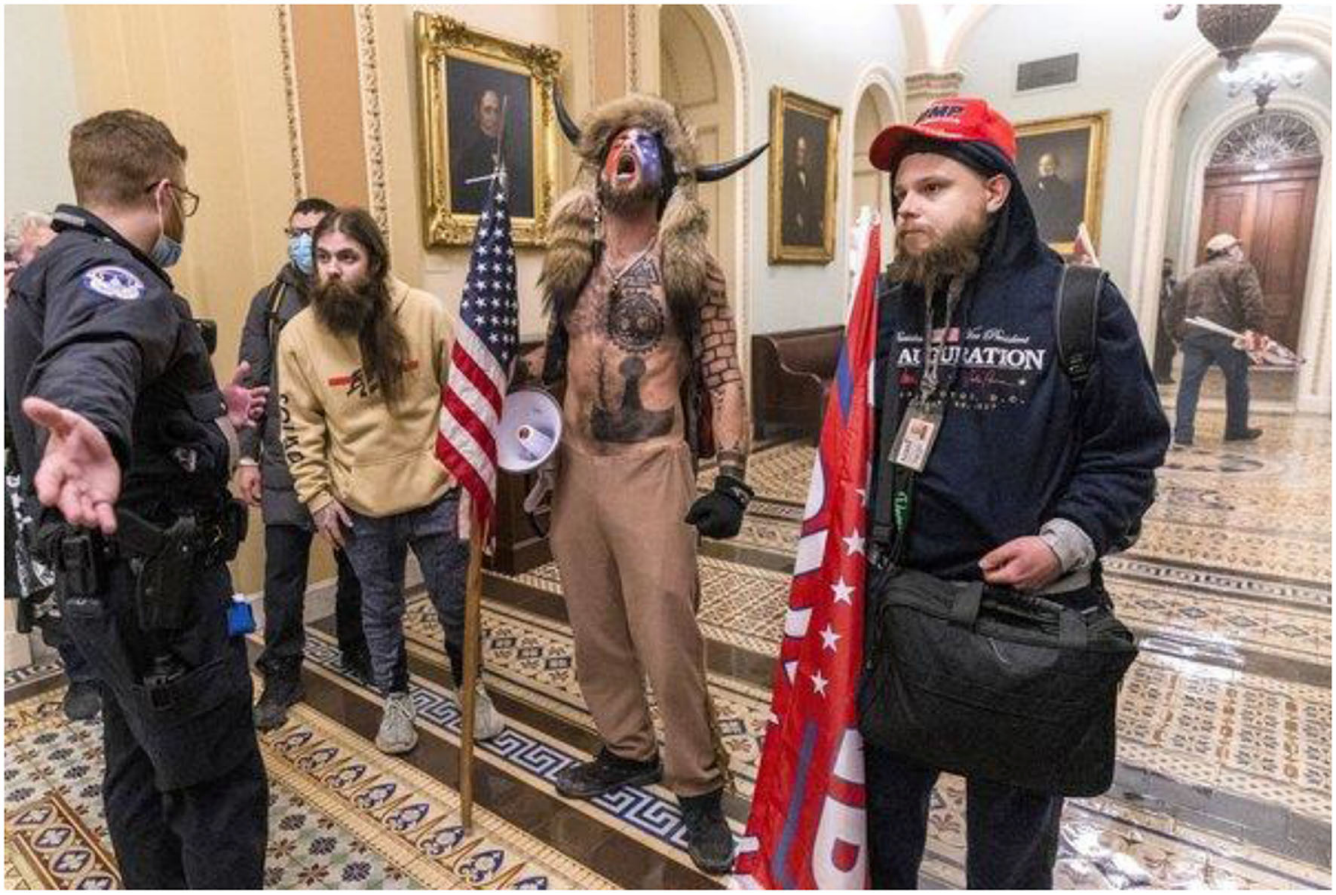
Jacob Chansley aka QAnon Shaman **(middle)** (Photo credit: AP Press https://apnews.com/article/capitol-riot-man-gets-organic-food-jail-198eccc02b8173f4e5071ca342250518).

My second indication that an alternative version of QAnon existed came from reading the two self-published books by Jacob Chansley (aka The QAnon Shaman, aka Jacob Angeli, see Figure 2). Methodologically, incorporating these books into this article could be seen as “life history method” in which I explore the lived experience of Angeli, through reading and analyzing his lived experience (Shaw, 1930; Blackman, 2021). In many ways Angeli reminded me of other occultists, neo-pagans, and other “new age” spiritualists I have encountered over the years. However, unlike those spiritualists, who generally were more “left” leaning, Angeli supported right-wing authoritarian figures. He also adhered to a conservative vision of the world which placed primacy on the centrality of the family, concern over sexual goings on of others, belief in psychic and other spiritual phenomena, other authoritarian traits discussed by Theodor Adorno and his contemporaries in *The Authoritarian Personality* (1950), and in the social psychological techniques used to illicit a response from their viewers.

As a researcher, and someone impacted by QAnon, I am also sympathetic to those who have been seduced by the peddlers of misinformation and see them as victims in an effort by some to profit off of misinformation.^7^ As Galliher (1980) has famously argued, sociologists ought to study and hold those individuals in powerful positions accountable. This would include those who would commit fraud on individuals, who would spread misinformation around life-saving medicines and vaccines, and those inciting violence against groups and governments.^8^ By studying up, as Galliher suggests, we avoid reifying knowledge on those most disadvantaged in society or who have been unfairly victimized. Thus, studying those at the top of this social movement may in fact shield those unfairly harmed by those seduced by these ideas.

## Other problems

Studying QAnon, especially in an era of COVID, has challenged many scholars—a point of consensus among scholars that I interact with. Sociology has historically been slow to adapt to ongoing societal changes and new technologies into research methods. These changes include incorporating online sites as nodes for qualitative research, the use of visuals (Harper, 2012), and attempts at work deemed overly political by gatekeepers (Salerno, 2022). This article illustrates the possibilities and affordances of observational research that can occur online. However, this kind of work is also met with enhanced skepticism that qualitative naturalistic observational research in the offline world does not always face. QAnon also problematizes existing research methods, due to its existence as an online phenomenon. Thus, this article makes an important contribution to queer criminology (Panfil, 2017, 2018) and queer methods (Ghaziani and Brimm, 2019), by showing the diversity that occurs on the ground level (see Blumer, 1969) and for researchers to look for innovative ways to conduct research—if for no other reason than protecting human subjects and the researcher from harm (i.e., contracting COVID).

## Research questions

Informed by the sociological literature, my research questions focused on the ways in which QAnon persisted in non-normative cultural spaces, especially those groups whose ideology seemed counterintuitive to QAnon. Specifically, I sought out to answer the following questions:

1. What does QAnon look like to those who engage in wellness groups, alternative spiritual practices, or other paranormal belief systems?
2. What kinds of authoritarian traits are exhibited by wellness promoters, alternative spiritual gurus, or through promoters of other paranormal ideas?
3. How does the work of the Frankfurt School, and the literature on authoritarianism, explain this seemingly bizarre mix of ideas?

In other words, the questions I asked sought to understand how seemingly incongruous ideas could be understood. I was also interested in seeing if this alternative version of QAnon reflected the same ideas, concepts, and techniques used by other “mainstream” promoters of QAnon.

### Findings

I organize my findings of this study into three major categories—Neo-Shamanism, Wellness/Yoga Instructors, and Psychics. Across each of these groups I found the presence of all of Adorno's nine personality traits. While I found examples for each of these traits, there were three that stood out to us as prominent across all the individuals we tracked. The most salient categories of Adorno's authoritarian personality scale were conventionalism (the rigid adherence to conventional, middle-class values), superstition and stereotypy (belief in mystical determinants of the individual's fate; disposition to think in rigid categories), and power and “toughness” (preoccupation with the dominance-submission, strong-weak, leader-follower dimension, identification with power-figures; overemphasis on conventionalized attributes of the ego; exaggerated assertion of strength and toughness).

## Neo-Shamanism, Starseeds, and other forms of alternative spiritual practices

Most individuals who watched the January 6th Capital riots, and who have made cursory attempts to find out more about QAnon, are familiar with the QAnon Shaman, so dubbed by CNN news anchors and other TV personalities. However, there is some truth in how he is described—Jacob Chansley, the QAnon Shaman, describes himself in his self-published book^9^ One *Mind At A Time: A Deep State of Illusion* (2020) as “an author, shamanic practitioner, a QAnon digital soldier, an energetic healer, a YouTube personality, a behavior health technician, a Navy Sailor, and a God-loving, country protecting patriot of the USA”. Within the pages of his text, he outlines a secret ruling elite that allow drug trafficking to occur so that they can have an untraceable money supply, accuses government agencies of engaging in espionage on American citizens, and calls out many other heinous acts committed by the government. In fact, a good chunk of his book is devoted to those things which most leftists criticize on a regular basis. However, slowly the book shifts from praising JFK to bizarre conspiracy theories and accusations that political elites engage in ritualistic “black magic” ceremonies. While in the book he never makes claims about Donald Trump, he does vilify high ranking Republicans and makes disparaging remarks about the Clinton Family.

Chansley's monograph is a near complete rehashing of the QAnon mythos without mentioning the anonymous poster or making direct reference to Donald Trump. This text, however, does give us insight into his conceptualization of himself as Shaman and his belief system:

There are really high vibratory fields and very low vibratory fields; however, they all exist within a certain perceivable frequency range native to this planet. Our five physical senses act like electromagnetic receivers which allow us to better navigate the narrow slice of the electromagnetic spectrum perceivable wall here in this world range of frequencies (85).

Here we are given a window onto Chansley's branch of Shamanism. His book is filled with the notion that all forms of life have a vibrational frequency that one can tap into, and that the mind through mental training or ritual can manipulate these frequencies. In interviews with journalists, Chansley says that yelling in the chamber was a shamanic practice meant to cleanse it from evil.

Chansley's book also contains several criticisms of society and ways in which he exercises praxis over his own life:

For example, to help put Monsanto out of business I have not bought any roundup weed killer, and I only buy organic, GMO-free produce and products when at the store. I have a list of all the Monsanto-owned subsidiaries and I do not buy from them either. I know this strategy will help to bankrupt evil corporations like Monsanto and other companies that are making profit by selling people poison; like alcohol and tobacco companies for example. However, one must still be cautious when buying food or other products, I have heard some organic brands like Kashi and Annie's were bought out by evil corporations, or have come under legal fire for lying about the substance of pesticides in their products. I do not consume pesticides because they have been linked to almost every type of cancer that we know of in the West as well as neurological disorders, IBS, and gastrointestinal diseases (116–117).

The above text illustrates how Chansley holds partially progressive views about Monsanto's ecological footprint and characterizes it as an evil corporation. These are all views you might even hear expressed in an introductory sociology course. Moreover, he takes it a step further by recognizing his own consumer power and in doing so realizes his agency. At the same time, however, his book suggests that, through engaging in a year of worldwide daily prayer, we can clean up the nuclear spill caused by TEPCO Corporation at the Fukushima nuclear power plant (Chansley, 2020, p. 49). We also start to see, from the quote above, the way conspiratorial thinking merges with his political ideology. One of the ways he does this is by viewing everything through the rigid categories of good vs. evil. Instead of going after a capitalist mode of production or larger structural issues he places blame on individual actors and companies. Secondly, he also seems to be alluding to some kind of hidden agenda, beyond making profits, by these corporations. While it is a bit more hidden in the above passage on page 70, for example, he discusses a network of tunnels running under the country—part of another aspect of the QAnon conspiracy movement which holds children are trafficked across the country using a network of underground tunnels.

**Figure 3:**
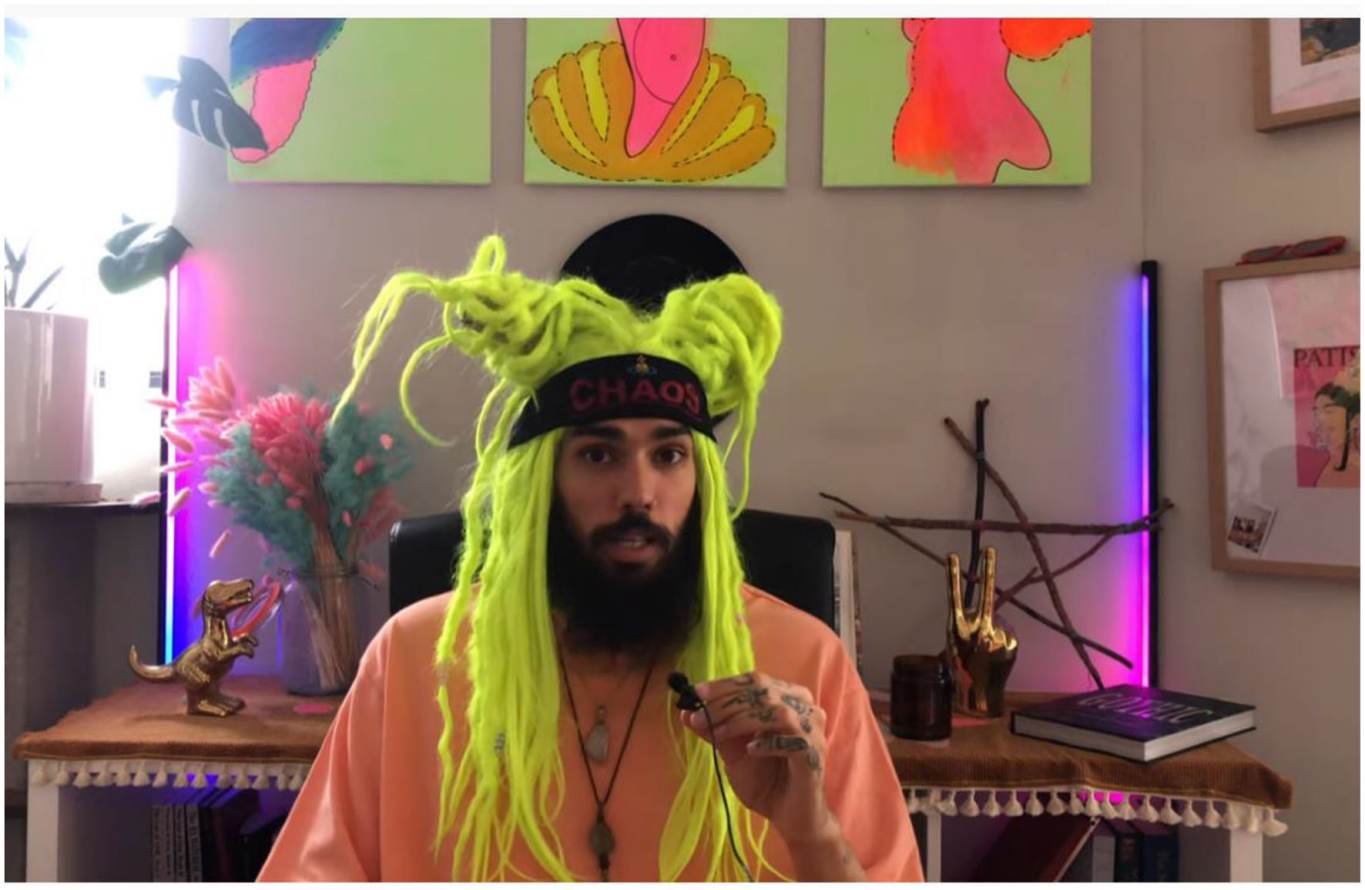
Akashic Daddy via Instagram.

If Chansley is the American distillation of QAnon and alternative forms of spirituality, then the person who goes by the name Akashic Daddy (AD) is his edgier Australian counterpart. AD wears a neon green wig and headband that says chaos (see Figure 3), and has also produced music for the gay club scene under the moniker Dad's Mayo. Chansley^10^ and AD also consider themselves “Starseeds”. A new form of spirituality, those who believe themselves to be Starseeds blend belief in UFOs, “New Age” Spirituality, and conspiratorial thinking. AD's videos on YouTube, podcast interviews that he has been a guest on, and other content gives people his theological insights; however, he also promotes a variety of COVID conspiracy and anti-vaccination/anti-mask sentiments (Crockford, 2021b).

In a recent podcast on which he appeared, he elaborated on his belief system.

My name is Akashic Daddy, nice to meet you. Yeah, no, so my name is Jay but I go by Akashic Daddy, I figured it sounds really funny. Like I don't know I feel like what happened to me is maybe two years ago I sort of like woke up to the fact that we're under this massive illusion, this full on global MK ultra situation, where most of the people are completely under the spell of—well now it's easy to say the media and Hollywood and all that but it's just like it's just so hard for that for people that don't know those things just sort of like pinpoint exactly what it is that's brain washing you… I need to just like get people to snap out of it like I can't deal being around people that are under the spell just like I can't really when I used to go out and party and when I was younger, I couldn't really stand being sober around people that were completely intoxicated… [sic]

His name, Akashic Daddy, is a clear reference to the Akashic masters who oversee a compendium of all universal thoughts, words, events, emotions, and intent that have ever occurred. In his interview he begins by talking about how the pandemic had revealed that Hollywood had placed everyone under a spell and that it is really a group of ruling elites controlling the world. Like conspiracy theorist Alex Jones, or David Icke, he repeats unfounded claims that this is part of an attempt to take rights and freedoms away from people. During the interview he reveals his connection to QAnon:

I was just trying to make like some like small pieces of content for every single thing that was on there [the Q map] and then with how my Instagram sort of like a bold it was I was I was just doing like I was just waiting for the news to happen and I will just do screenshots at the news and just tweet like making comments about it. [sic]

**Figure 4:**
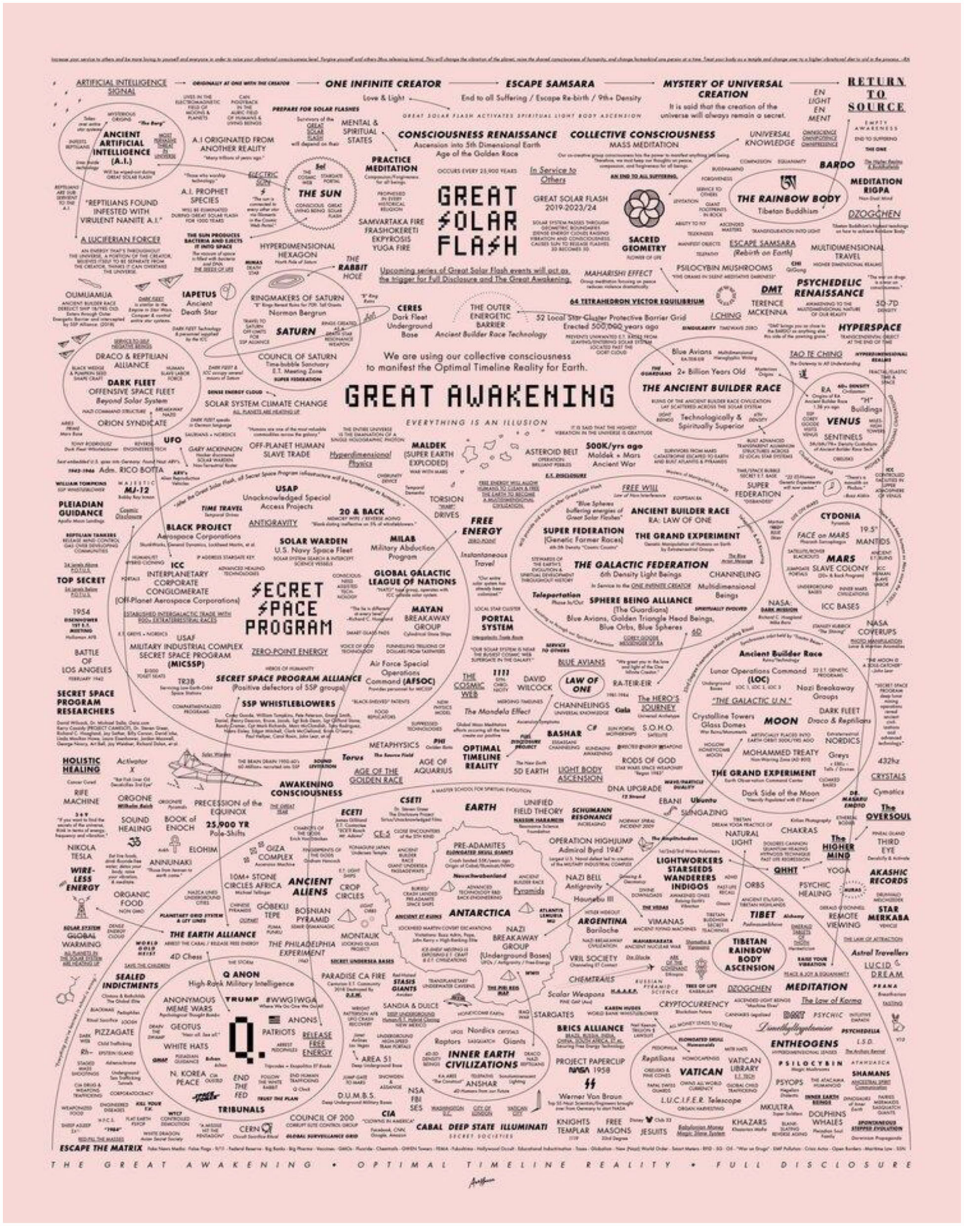
The QMap from QMap.Pub.

The QMap is an image that contains a variety of conspiracy theories, at the center of which are the words “The Great Awakening” (see Figure 4). In the QAnon Universe, the great awakening is a moment in which all non-believers will be woken up by the truth—giving the movement a directive to proselytize to others and “spread the good word”. There are moments, like with Chansley, where he begins to criticize government leadership during the pandemic:

…there's still so many people on both sides and anti vax debate. There's people on both sides pointing the fingers, and some saying “no you're stupid because of this”. When I went to the answer them, I suggested that both sides need to merge and that if any fingers be pointed, it should only be toward the freaking government. [sic]

While AD correctly identifies the government and other institutions that are failing their citizenry, it's his words between those moments which are verifiably wrong. I point this particular one out to illustrate the contradiction that exists between his societal critique, and his embracement of authoritarian leaders—namely Donald Trump. Whether or not he is aware of the anti-Semitic undertones in some of his beliefs is unknown, which is part of the real danger the larger QAnon movement holds—it mainstreams hate speech by placing a spectacular veneer over it.

## Wellness, fitness, and yoga instructors

Among the most surprising groups for us, at least initially, for QAnon to appear within were wellness, fitness, and yoga instructors. A wide range of reporting and research has been done on this area (see Ward and Voas, 2011; Nelson, 2021), including a special segment aired on comedy central's news show “The Daily Show”. As I began diving into this area, I found more and more people that were also promoting ideas found in QAnon—this includes anti-vax and COVID misinformation, political misinformation regarding the election, and support for Donald Trump. As investigative journalists have reported, unlike other tactics used by QAnon promoters, this “soft” version of QAnon helped draw in those who were somewhat unsuspecting. This includes those wanting to get more physically fit, those seeking alternative forms of therapy when modern medicine has failed them, and still for others a search for both a community of likeminded individuals and a spirituality that resonated with them. As I will return to in the discussion, there is a thread of narcissism that runs throughout the wellness influencers who promote QAnon.

One of the most prominent figures within the wellness industry to QAnon pipeline is Troy Casey, the Certified Health Nut. Casey's website, YouTube channel, and other sites contain a lot of information for free. However, Casey also offers an array of services and products ranging from online courses in Wim Hoff breathing, access to his Man Clan fitness group, his book *Ripped at 50*, and supplements produced by Purium for which he is in affiliate salesperson. However, underlying this is a whole set of ideologies which, as we shall discuss later, reinforce gendered stereotypes about masculinity and promote a far-right political ideology. It is not uncommon for Casey to bring up essentialist ideas when he discusses race, gender, or even sexuality. While some of his fitness information is rooted in proven methods, just as much, if not more, is rooted in pseudo-science and contains potentially harmful advice.

Like other influencers, much of Casey's life is documented online. I found a podcast in which he was a guest on the topic of biohacking and alternative modalities of wellness. On the podcast with Casey is another wellness influencer, the self-proclaimed “Sikh Kundalini Yoga Guy” Har Hari. Below is an excerpt from their discussion:

**Har Hari:** The first person who got the backlash was a Sikh in Arizona. He got killed literally right after 9/11… And there was a big massacre, I think, in Wisconsin a few years ago. Same thing, the guy is a freaking dumb ass redneck, I want to kill those Muslims. He doesn't even know what the difference is between a Muslim and Sikh. And he goes in there with a gun and just massacres them. Yeah. I mean, it's scary, means the level of ignorance is, oh, my God. It's scary…

Here we are given a potentially progressive stance and analysis of 9/11. However, this is quickly undermined with their discussion of the role of the beard among the Sikh, Navajo, and other ethnic groups. They note the masculine power and strength that they both feel from their beards and hair before moving on.

**Har Hari:** Yeah. I mean, the hair is, the Indians, I'm talking about the Native Americans, they knew about hair. There was a story in World War II where they got the guides. And when they cut their hair, they lost their skill.

**Troy Casey:** Their tracking skills.

**Har Hari:** Their tracking skills were gone. So, the hair is like an antenna. Basically, it acts like an antenna, when you wrap it in a bun on the top of your head, it's like channeling the cosmic energy through your 10th gate, through your crown chakra. And especially for men having hair on your face, it's actually insulating you from the moon, the energy of the moon. Men are not supposed to be influenced by the moon. Women are because they have a cycle. But men are just connected with the sun, basically.

**Troy Casey:** So, yeah, I heard that the beard protects you against the feminine charms. [sic]

In the above exchange, Casey and Hari simultaneously reinforce forms of toxic masculinity and promote racialized stereotypes of particular racial and ethnic groups (Said, 1979; Han, 2015; Nguyen, 2021). The above exchange also illustrates their rigid views of masculinity and gender—where masculine is seen as a positive force and the feminine is seen as something to be feared. The interview then quickly shifts, and we start to get a glimpse into how their fitness ties into their political ideology as the quote below illustrates:

**Troy Casey:** And I've noticed that I have a lot more respect. Now, there's a lot of people in the debt slave system. They're like, Troy, you should shave and you'll make more money. And the funny thing is, is I had the ultimate classic look as an actor and a model for so many years. And I had some success for sure, but it wasn't the major success that I wanted. And it wasn't until I grew up my beard that everything changed. People treated me differently because, even though I was approaching 50, in my 40s, people still treated me like a kid. I don't know what it was about my energy. And I'm the same person, same personality. But now, when I have the beard, people give me more respect. I'm treated completely different, more like an elder.

Again, the above quote illustrates the signs of toxic masculinity that underlie all kinds of aspects about how he lives his life, wrapped in a kind of neo-shamanism and quest for timeless, old, and discarded knowledge by which they can return. But, more importantly, they are trying to impress upon their listeners that they hold some kind of hidden knowledge to unlock the secrets of life. The interview then pivots once more into conspiracy theories related to COVID-19 and QAnon.

**Troy Casey:** I've heard the inklings. I don't follow every nuance of what he's doing. I follow the QAnon post because I really like those and pray that there is going to be a big shift. But the one thing that I noticed, and it came in his Twitter was the Covfefe thing. You want to think about that?

**Luke Storey:** Yeah. What was that?

**Troy Casey:** The Covfefe thing-

**Luke Storey:** I thought he mispronounced coffee or something.

**Troy Casey:** So, I've heard recently that that is the antidote for 5G ionization because it's cobalt [CO], FE is iron, right? So, two iron molecules and a cobalt, and it protects against the ionization, something like that. Look into it. I don't know. [sic]

While these figures may seem like fringe figures, and perhaps that is true, it is important to note here that there are many individuals within the wellness community promoting these ideas. These ideas are so popular that they have appeared recently on Fox News, Joe Rogan, and other promoters and platforms with massive user bases. Together these figures make up part of what is referred to colloquially as the “manosphere”, which is a collection of online groups that promote the idea that feminism is ruining the country (Baker, 2017). Figures within the manosphere, like Troy Casey, combine body building information and other wellness techniques while also injecting his audience with misinformation and political ideology. Another example of this is taken from Troy's Instagram account:

What do you think about all this “inclusion” propaganda and social engineering? Do you think I'm spreading “hate” by asking deeper philosophical questions? What does this say about @caitlynjenner as he/she was decathlon champion and I do believe the 1976 Olympics! [sic]

Here Troy is responding to a tweet made by Caitlyn Jenner that reads “I don't think biological boys should compete in women's sports.” In Troy's world, where nature and natural law are supreme, we see how those views coalesce with his political ideology and eventually bring him to his ultimately conservative position.

## Numerologists and other practitioners of magic

The final group of individuals that I found within the alternative QAnon belief eco-system were numerologists, psychics, and other practitioners of magic. Like those who engage in alternative practices within spiritualty circles, those who I put into this group believed they could harness some hidden practice to change the world around them—either some kind of “6th” sense, use of divine knowledge of numbers, or some kind of other mystic art (tarot cards, ritual, etc.). It was also the least pronounced of the others I monitored and tracked. This is likely due to the QAnon movement wanting to distance itself from any form of what might be called “magic”, because those are the tools of “the deep state”. On the other hand, numerology appears throughout all of QAnon either as a divine message, cryptographic tool used by Q or Donald Trump to convey hidden meaning, or as an indicator of some other divine presence.

Among those that I monitored closely, Brian Protzman, also known as Negative 48, was among the most prominent figures. Protzman became infamous for encouraging his followers to go down to Dallas Texas to await the resurrection of John F. Kennedy—when he didn't show up, they stayed. Since November 22, 2021 the group following Negative 48 have remained in Dallas faithfully adhering to the commands of Protzman and presumably awaiting the return of JFK. According to QAnon researchers who have infiltrated the group, he has convinced many of his followers to turn over their life saving to fund the group's activities and engage in the drinking of bleach (Murney, 2021; Rohrlich, 2021). Since his emergence, Protzman has closed himself off to those outside of his group by living in a hotel in Dallas—presumably using the funds given by his followers to pay for their continued stay.

**Figure 5:**
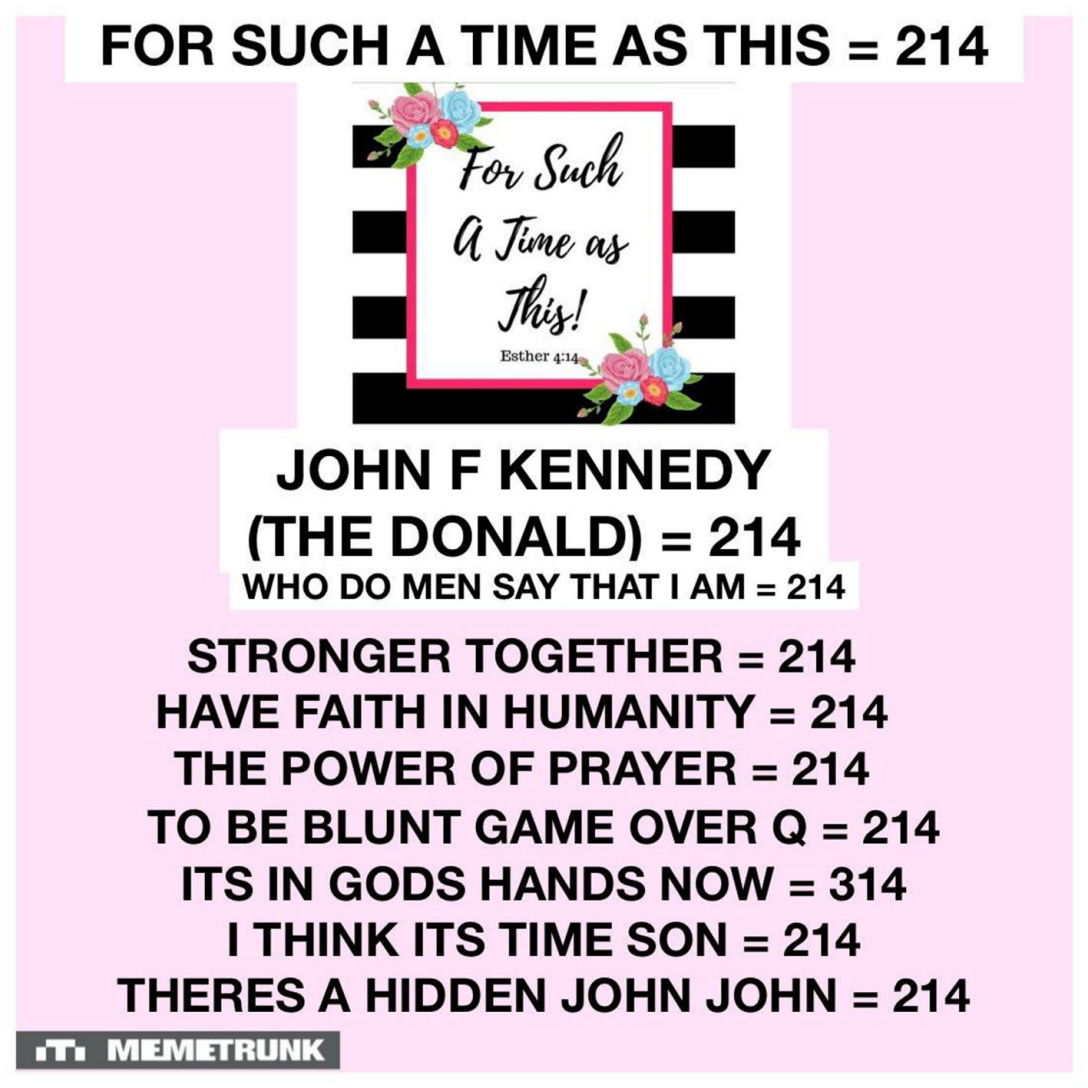
Example of gematria by a “Proud Protzmanian”.

Protzman represents a group of individuals who practice a kind of “folk” version of numerology known as gematria. Figure 5, posted by one of his followers, illustrates the process by which devotees translate words into numbers to find meaning. First, they assign the words a numeric value and then they look for similar words of phrases that also have the same numerical value. If they do, then they are somehow linked.

The image above seems to be trying to make the connection that Donald Trump and JFK are one and the same. This is because this particular branch of QAnon followers believe that JFK is in fact still alive, and they revere him as the last great President. Here I see how they try to link present day happenings with their worldview.

Most practitioners of this form of divination do not necessarily make claims, but rather do the math and let their audiences make the connection. Most practitioners of gematria when practicing, or creating online content, speak in ways that make little sense. This is because they simply put the “evidence” out there and move on to the next topic. One such practitioner, who gained notoriety because of an endorsement by comedian and actress Roseanne Barr, is a man who goes by the name Tom Numbers. He has made himself into something of a unique QAnon influencer in that he uses numerology to decode larger historical happenings, and also to justify the prophecies of Q. He also used to appear with Brian Protzman in video content. The below is an example of a reading he has given online and posted to a streaming website:

So Friday the 13th comes to 223, Friday is 63, it always makes me think of the year 63 with JFK. Also the word eleven when you spell it out is 63, but the day Friday is 63. Verse 33, 13th is 127 which is the ace of spades, which is magic November, magic john john, it comes to 223, 223 comes to Global Currency Reset. And next year we are going into 2023. One of the ways Juan likes to do the numbers is to drop the 0s so that would be 223. I looked at it another way. If we do Friday the 13th, and if you just do one + three and then the TH. So 13 plus TH comes to 41, 41 comes to King, comes to Alien, comes to USA, comes to Good, comes to Fun, comes to my initials TSB…[sic]

While his decoding here doesn't quite point to QAnon, it does provide an example of his technique. First, he assigns a value to letters in a particular word, then adds those to any numbers, and then it produces a result. If two words have the same numerical value, then they are believed to be “cosmically aligned”. In the case of the reading above, Numbers is trying to create a connection with Friday the 13th (the date of the recording) and other terms, however he never fully elaborates on that connection. He also re-calculates his numbers using a different method, which to a critical observer calls into question his method of divination.

## Discussion

While it may seem unfathomable that a right-wing conspiracy theory could take off in spaces organized around improving health, finding spiritual meaning, or looking for answers from the great beyond, these groups may provide fertile ground for conspiracy theories to spread. My first research question sought to understand the aesthetic form of these different groups. What I found was on the surface they looked like any other social media influencer in their respective genres. The danger here is that someone attempting to learn about self-improvement may end up inadvertently being convinced of extreme right-wing views about global ruling elites—which is precisely what others have already found (Crockford, 2021b; Buntain et al., 2022).

Part of this stems from Barkun's (2013) observation that stigmatized knowledge tends to occupy the same spot, and thus tends to feed off other forms of stigmatized knowledge—a finding echoed by those who study the spread of far-right ideologies in online spaces (Beran, 2019). However, Crockford's (2021a,b) work points to the Americanization of “Eastern” philosophy as a driving mechanism for how and why these ideas spread in alternative spaces. As I have shown here, these ideas are also the byproduct of the authoritarian traits of superstitious and mystical thinking (the first authoritarian trait), which are used to reify and promote conventionalism (second authoritarian trait), and power and “toughness”, which are used as symbols of success that continue to drive individuals further into these belief systems (Rosen, 2019; Crockford, 2021a,b).

Secondly, I sought to understand the kinds of authoritarian traits exhibited by the groups studied herein. What I found was that this new form of spirituality has no allegiances to building community and is one deeply rooted in individualism—both exemplifying rhetoric of the American Dream and the influence of social media and celebrity culture which increasingly isolates and orients one toward hyper-individualism (Turkle, 2011; Rojek, 2016). Like a Trojan horse, far-right groups use these seemingly benign, if not liberal, cultural phenomenon to hide their embedded far-right messaging.^11^ Through this tactic, they are able to push authoritarian/fascist narratives and make them appeal to massive audiences. Appealing to larger and larger audiences is thus not only important for the promotion of the movement's ideology, but also for financial support necessary to keep them alive (Miller-Idriss, 2020).

The role of the internet in the spread of QAnon and right-wing extremism has increasingly been a matter of scholarly and public discourse (Nagle, 2017; Beran, 2019; Forberg, 2021). The work of the Frankfurt School is well situated to unpack and understand the phenomenon I've analyzed herein. The concepts of the culture industry, first introduced by Horkheimer and Adorno (1972 [1944]), argued that Fordist-style production techniques were increasingly being used to mass market products. Like the radio, television, and movies in their time, the technological innovations that have given birth to the internet, social media, and content creation allow for the implementation of Fordist-style production techniques on an unparalleled scale. Additionally, sites like YouTube incentivize influencers and content creators to produce material. The actors who promote these ideas utilize the same tactics other internet entrepreneurs engage in, and are rewarded for their efforts through the monetization that these platforms provide. Simply put, attracting more followers means more funding, and this in turn leads to further spread of these ideologies. Thus, these “influencers” are incentivized to create right-wing content that is appealing, extreme, attractive, and entertaining to audiences—many of whom may be looking for answers for problems in their own lives only to stumble into a right-wing rabbit hole.

Thus, the tactics employed by agents of the culture industry are also utilized here to strip these groups of their politically dissident features in pursuit of profitability. In turn, these pursuits uphold existing power dynamics and promote nationalist discourses (Horkheimer and Adorno, 1972 [1944]). In so doing, far-right groups have been given freedom to exist, attract new members, and grow their power and influence (Wylie, 2019). As social media and the internet continue to be commodified spaces of public interaction and discourse, the companies continue to organize them in ways which maximize their profits—often at the expense of the public. Just as popular cultural influencers spread the latest jokes, memes, and trivia, so too have far-right cultural influencers emerged in this larger echo system (Forberg, 2021). Moreover, this kind of subcultural appropriation reminds us of the problems with the commodification of culture—in the service of capitalism they lose their emancipatory potential (Marcuse, 1972).

## Conclusion

Among the January 6th rioters on the capitol were individuals wearing counter-cultural clothing that a decade earlier would have signaled, even superficially, an alignment with liberal ideology. This purpose of this paper has been to draw attention to the spread of authoritarian and fascist tendencies within non-stereotypical cultural arenas. In this study I have looked at alternative spirituality, but in the course of my observations I was made aware of a variety of other strands of authoritarian right-wing ideology embedded in space that might be overlooked by the casual observer—including MMA sports, music subcultures, and a range of other conspiracists (Enders et al., 2022). As scholarship continues to emerge on the lasting impacts of conspiracy movements like QAnon, future research is necessary to properly document these strands as researchers attempt to resolve the issues created in their wake.

Journalists such as Yunkaporta (2022) and scholars such as Victor (1998) remind us that conspiracy theories and alternative belief systems can lead to horrific human rights abuses—as was the case in Australia when the military was mobilized toward aboriginal communities under false accusations of child trafficking. In the United States, we have seen individuals commit heinous acts in the name of QAnon, which holds similarities to the Satanic panic of the 1980s (see Victor, 1998). However, as Barkun (2013) argues, there may be something inherent in American culture as a plethora of examples exist going as far back to the late 1950s (Dohrman, 1958) in which the promotion of racism and xenophobia were used to “save the children”.

While conspiracy theories and authoritarianism has been analyzed from a right-wing perspective, as I have shown here, authoritarianism can occur, however unlikely it may seem, in spaces which appear more culturally progressive. It is within these unlikely spaces that we must pay attention as these ideas have the ability to cross over into the mainstream and inadvertently promote extremist ideas through alternative mechanisms. The simple practice of going to a Yoga class, or a wellness coach, may provide tangible health benefits but may also promote pseudo-scientific, dangerous medical misinformation, or justify existing xenophobic ideas (i.e., racism, sexism, homophobia, or transphobia). Already we have seen this happen with podcasters and “pseudo-academics”, many who have audiences in the millions (Conner and MacMurray, 2022). At the very least this kind of spiritualism tries to justify neo-liberal policies that contribute to growing wealth inequality, climate change, and a whole host of other social issues.

Finally, the connection between the rise in social media and extremist movements and conspiracy theories is noteworthy. As others have begun to document, the algorithms used by tech companies have played a significant role in breathing life into QAnon and extremist content (O'Neil, 2016; Wong, 2020). New computer technologies have opened up endless possibilities for reconfiguring human social interaction (Kellner, 1989). To date, however, new computing and communications technologies have been used primarily in ways that increase profit at the cost of elements of the human experience. These qualities, some argue, are the qualities which make life worth living—community, creativity, compassion, and even love. In order to save the things that we agree are human qualities worth keeping, we may have to find mutual points of interest by which we can create productive dialogue among individuals with competing narratives about reality rather than relying on technology to solve these problems.

## Data availability statement

The original contributions presented in the study are included in the article/supplementary material, further inquiries can be directed to the corresponding author.

## Ethics statement

The Institutional Review Board of University of Missouri—Columbia waived the requirement for ethical approval. Written informed consent was not required for participation or the publication of potentially identifiable data in accordance with the national legislation and the institutional requirements. The social media data was accessed and analyzed in accordance with the platforms' terms of use and all regional/institutional requirements.

## Author contributions

The author confirms being the sole contributor of this work and has approved it for publication.

## Footnotes

^1^Live streaming and other new forms of content relationships allow for the producer and consumer to interact in a way that is unique. While Adorno and Lowenthal were writing about the radio, livestreaming carries with it the same processes, but introduces an interactive chat so viewers can react in real time to the content produced.

^2^The Institutional Review Board of University of Missouri—Columbia waived the requirement for ethical approval. Written informed consent was not required for participation or the publication of potentially identifiable data in accordance with the national legislation and the institutional requirements. This is because the University IRB board did not consider this research with human subjects since my data is based on observing online public behavior. In my analysis, however, I do my best to present my subjects in the best possible light.

^3^While some may argue this is a poor substitute for ethnographic research, both the research protocols put in place during the COVID-19 pandemic and the distrust by adherents to the movement made close observation problematic. We approached multiple QAnon event organizers and were denied entry to events, those attending the events that we contacted refused to contact us, and we were generally confounded by my attempts to do formal interviews with participants. This likely stems from their distrust in traditional institutions and the underlying QAnon philosophy which holds that researchers like us were deep state operatives. Thus, watching from afar in an observational style (Draus et al., 2005, p. 171) was the next best thing we could do along with content analysis of media created by QAnon influencers. Without proper multi-year funding, finding recruitment of subjects for such a study will continue to be problematic (Draus et al., 2005).

^4^To use the language of contemporary scholars, this was a convenience-based sample.

^5^An example of this is Tim Pool, an American political commentator and podcast host who has given a platform to Neo-Nazis and other shadowy cultural figures (O'Connor, 2023).

^6^I'm very appreciative of a colleague, Nicholas MacMurray at Rockhurst University, in drawing this to my attention, to three undergraduates Samir Knox, John Callendra, and Ronney Phillips for their help in data collection, and to QOrigins and The QAnon Anonymous podcast. Finally, I am thankful for the late David R. Dickens who reviewed an early version of this paper.

^7^An argument raised by the lawyers representing the QAnon Shaman in his trial.

^8^Often, in an effort to profit. As was the case made against talk show host Alex Jones and his recent trial.

^9^Published under the pen name Jacob Angeli (aka Jacob Chansley, aka The QAnon Shaman).

^10^His now deleted YouTube channel was titled “Starseed Academy”. Also, this connection is not to imply that all those who consider themselves Star seeds are part of QAnon, just that some overlap exists.

^11^This strategy is so common that members of far-right organizations call it “hiding your power level”. Instead of saying overtly racist ideas they try to hide them with dog whistles, the tactical deployment of irony and satire, or through elaborate qualifying statements.
